# Edible bird’s nest ameliorates oxidative stress-induced apoptosis in SH-SY5Y human neuroblastoma cells

**DOI:** 10.1186/1472-6882-14-391

**Published:** 2014-10-13

**Authors:** Mei Yeng Yew, Rhun Yian Koh, Soi Moi Chye, Iekhsan Othman, Khuen Yen Ng

**Affiliations:** Jeffrey Cheah School of Medicine & Health Sciences, Monash University Malaysia, Kragujevac, Selangor Malaysia; Department of Human Biology, School of Medicine, International Medical University, Kuala Lumpur, Malaysia

**Keywords:** Edible bird’s nest, Apoptosis, SH-SY5Y, 6-OHDA, Neurodegenerative disorder, Parkinson’s disease, Neuroprotection

## Abstract

**Background:**

Parkinson’s disease (PD) is the second most common neurodegenerative disorder affecting the senile population with manifestation of motor disability and cognitive impairment. Reactive oxygen species (ROS) is implicated in the progression of oxidative stress-related apoptosis and cell death of the midbrain dopaminergic neurons. Its interplay with mitochondrial functionality constitutes an important aspect of neuronal survival in the perspective of PD. Edible bird’s nest (EBN) is an animal-derived natural food product made of saliva secreted by swiftlets from the *Aerodamus* genus. It contains bioactive compounds which might confer neuroprotective effects to the neurons. Hence this study aims to investigate the neuroprotective effect of EBN extracts in the neurotoxin-induced *in vitro* PD model.

**Methods:**

EBN was first prepared into pancreatin-digested crude extract and water extract. *In vitro* PD model was generated by exposing SH-SY5Y cells to neurotoxin 6-hydroxydopamine (6-OHDA). Cytotoxicity of the extracts on SH-SY5Y cells was tested using MTT assay. Then, microscopic morphological and nuclear examination, cell viability test and ROS assay were performed to assess the protective effect of EBN extracts against 6-OHDA-induced cellular injury. Apoptotic event was later analysed with Annexin V-propidium iodide flow cytometry. To understand whether the mechanism underlying the neuroprotective effect of EBN was mediated via mitochondrial or caspase-dependent pathway, mitochondrial membrane potential (MMP) measurement and caspase-3 quantification were carried out.

**Results:**

Cytotoxicity results showed that crude EBN extract did not cause SH-SY5Y cell death at concentrations up to 75 μg/ml while the maximum non-toxic dose (MNTD) of water extract was double of that of crude extract. Morphological observation and nuclear staining suggested that EBN treatment reduced the level of 6-OHDA-induced apoptotic changes in SH-SY5Y cells. MTT study further confirmed that cell viability was better improved with crude EBN extract. However, water extract exhibited higher efficacy in ameliorating ROS build up, early apoptotic membrane phosphatidylserine externalization as well as inhibition of caspase-3 cleavage. None of the EBN treatment had any effect on MMP.

**Conclusions:**

Current findings suggest that EBN extracts might confer neuroprotective effect against 6-OHDA-induced degeneration of dopaminergic neurons, particularly through inhibition of apoptosis. Thus EBN may be a viable nutraceutical option to protect against oxidative stress-related neurodegenerative disorders such as PD.

## Background

Parkinson’s disease (PD) is an age-related progressive neurodegenerative disease with estimated worldwide prevalence approaching 9 million of people over the age of 50 by 2030 [1]. Pathologically, there is loss of dopaminergic neurons in the substantia nigra which subsequently causes dopamine depletion in the striatum [2]. Abnormal aggregation of α-synuclein known as Lewy bodies is also detected in surviving neurons [3]. Dopamine depletion ultimately leads to deterioration of motor functions whereby the patients are often manifested with clinical signs such as tremor, rigidity and slow responsiveness [4].

Several hypotheses including neuroinflammation, mitochondrial dysfunction, failure of ubiquitin-proteasome system and proteinopathy have been proposed to explain the neurodegeneration events in PD [5, 6]. Amongst those, oxidative stress-related apoptosis has been implicated in the pathogenesis of neurodegenerative diseases. Oxidative stress is caused by the production and accumulation of excessive partially reduced reactive oxygen species (ROS) within the cell, which attacks electron-rich biological molecules such as DNA, protein and lipid to affect cellular functions [7]. ROS is generated as a part of normal cellular metabolism. In the cells with high oxygen-utilizing capacity such as the neurons, however, greater amount of highly reactive oxygen radical is being produced which renders these cells more vulnerable to oxidative damages [8]. In PD, substantial post-mortem studies noted that impaired mitochondrial function and ROS build up are two events linked to apoptotic episode in dopaminergic neurons [6, 9]. Experimental models of PD which are generated through the use of mitochondrial complex I activity-inhibiting and ROS-inducing neurotoxins are able to recapitulate the pathological features in PD, further reinforces that ROS introduction is critically involved in the disease [10]. Therefore the brain requires an effective antioxidant system to counteract the impact of ROS, as well as an anti-apoptotic mechanism to maintain the neuronal integrity.

Edible bird nest (EBN) is natural food product made from saliva of the swiftlets of the genus *Aerodramus* (or *Collocalia).* Numerous *in vitro* and *in vivo* researches have shown that administration of EBN was able to boost immunity, promote cell division and proliferation, neutralize influenza activity as well as improve osteoporosis [11–14]. Studies have shown that EBN contains the bioactive compound sialic acid [15–17]. Furthermore, EBN may also contain epidermal growth factor (EGF) because EGF-like activity was detected in protein fractions partially purified from EBN extract. In fact, sialic acid and EGF are neurotrophic factors known to promote neuron and brain development [18–21]. On the other hand, animal saliva was previously found to contain vascular endothelial growth factor and melatonin [22, 23]. These compounds are powered with anti-apoptotic and antioxidant properties [24, 25]. As apoptosis and oxidative stress have been suggested as crucial events in neurodegeneration, EBN, the salivary secretion of swiftlets, may have neuroprotective relevance in the therapeutic context of PD. Nevertheless no scientific investigation has been conducted thus far to confirm this. Hence this study aimed to investigate the neuroprotective effect of EBN.

## Methods

### Preparation of EBN extracts

Raw EBN from the swiftlet of *Aerodamus* genus collected from bird’s nest farm in Perak, Malaysia was kindly provided by a local EBN distributor Yew Kee Pte Ltd. Cleaning was carried out by first soaking the unprocessed EBN in ultrapure water until softened and protein strands became slightly loosened. Dirt and feathers were removed manually by forceps. Cleaned EBN was subsequently oven-dried at 50°C before being grounded into fine powder. A portion of cleaned EBN was kept for water extraction whereby the EBN was first soaked in cold distilled water for 48 hours followed by boiling at 100°C for 30 minutes. The solution mixture was filtered and the filtrate was freeze-dried with freeze dryer (EYELA Freeze Dryer FOU 2100) to obtain EBN water extract powder.

Traditionally, a bird’s nest soup was prepared by double-boiling the cleaned EBN strands with water until softened, whereby sugar is often added to enrich the taste. In the current study, however, both raw EBN and its water extracts were prepared by enzymatic digestion using method adopted from Guo *et al.* [13]. Pancreatin digestion was performed as numerous studies have suggested that proteolytic breakdown of EBN produced greater bioactivities when compared to the undigested EBN [13, 14]. This additional step of enzymatic hydrolysis is suggested to enhance solubilisation of bioactive compounds, which subsequently leading to cellular assimilation. Briefly, raw EBN and water extract powder dissolved in ultrapure water at 2.5% (w/v) were digested with pancreatin (final concentration 0.5 mg/ml) (Sigma Aldrich, USA) in a 45°C water bath for 4 hours at pH 8.5- 9.0. Pancreatin enzyme was then deactivated at 90°C for 5 minutes. The mixtures were then filtered and freeze-dried to obtain the final crude and water EBN extracts, which were denoted as S1 and S2 respectively. Finally, products were dissolved in dimethyl sulphoxide (DMSO) (Sigma Aldrich, USA) as a stock of 50 mg/ml and sonicated until the powder was fully solubilized. Then the EBN solutions were centrifuged at 3000 rpm for 10 minutes to precipitate the undissolved EBN particles. The supernatant was collected and stored at -20°C for future use.

### Neuronal cell culture

Human neuroblastoma cells SH-SY5Y was purchased from the American Type Culture Collection (ATCC no. CRL-2266) and cultured in complete medium prepared from Dulbecco’s Modified Eagle’s Medium (Gibco, UK) supplemented with 10% fetal bovine serum (Gibco, UK). The cells were maintained at 37°C humidified incubator with 5% CO_2_ for 2-3 days until 70% confluent. Cell collection was carried out by rinsing the cells with phosphate-buffered saline (PBS) (Biobasic, Canada) followed by addition of trypsin-EDTA (Gibco, UK) to detach the cells. The action of trypsin was later neutralized with complete medium and cells were harvested by centrifugation at 1500 rpm for 5 minutes. The cells were then sub-cultured into new tissue culture flask or plated for assays.

### Determination of maximum non-toxic dose (MNTD) and effect of the EBN extracts on 6-OHDA-induced cytotoxicity

Cytotoxic test was performed with tetrazolium reduction assay using 3-(4, 5-dimethylthiazol-2-yl)-2, 5-diphenyltetrazolium bromide (MTT) reagent (Sigma Aldrich, USA). Cells were first seeded onto 96-well plate at a density of 4 × 10^4^ cells/well with complete medium, which then was replaced by serum-free medium for treatment in the next day. Cytotoxic effect of both crude and water EBN extracts on SH-SY5Y cells was tested across a wide range of concentrations from 0 to 500 μg/ml. DMSO, which was used to dissolve the extracts, was included as vehicle control. After 48 hours incubation, MTT solution was added into the culture to a final concentration of 0.5 mg/ml. After 4 hours incubation at 37°C, the medium was removed and replaced with equal volume of DMSO to dissolve the purple formazan crystal. Absorbance of the solution was measured spectrophotometrically with microplate reader (DynexOpsys MR 24100) at 570 nm and was compared to control to be presented in percentage of cell viability or toxicity. MNTD and ½ MNTD of EBN extracts were determined from graph plotted.

To determine the effect of EBN extracts on SH-SY5Y intoxicated with neurotoxin, cells were pre-treated with EBN extracts at MNTD and ½ MNTD for 24 hours followed by co-incubation with 100 μM 6-OHDA for another 24 hours. Upon completion of treatment, MTT assay was performed to determine the cell viability. All test assays followed the same treatment whereby DMSO alone (0.5% v/v) was used as vehicle control.

### Morphological examination

Apoptotic cells experiencing damage in the nuclei are featured by cell shrinkage, membrane blebbing and presence of apoptotic bodies [26]. In order to perform morphological study, cells were first grown in 60 mm culture dish and treated accordingly whereby groups such as vehicle control, 6-OHDA, S1 MNTD + 6-OHDA and S2 MNTD + 6-OHDA were included. Then, cell morphology was examined under bright field inverted microscope (Nikon Eclipse T*i,* Japan). In addition to that, nuclear staining was performed with Hoechst staining. Treated cells were fixed with 4% paraformaldehyde for 15 minutes before stained with Hoechst 33258 (1 μg/ml) (Sigma Aldrich, USA) for 15-20 minutes. Nuclear changes were examined under fluorescence excitation using the same microscope for features such as chromatin condensation, DNA fragmentation and cell shrinkage. Photomicrographs were taken using attaching camera.

### Intracellular reactive oxygen species (ROS) level measurement

Intracellular ROS production was assessed with 2′, 7′-dichlorofluorescein diacetate (DCFH-DA) fluorescent probe. Cells were seeded into 12-well plate at a density of 1.5 × 10^5^ cells/well. Upon completion of treatment, cells were collected and washed before added with 40 μM DCFH-DA (Sigma Aldrich, USA) working solution in 96-well black plate. Fluorescence reading was taken at 0, 10, 20 and 30 minutes with fluorescence microplate reader using excitation and emission wavelengths of 485 nm and 535 nm (Tecan, Austria). The fluorescence readings were then normalized to the respective cell number to give relative value of DCF fluorescence unit. Fold change in ROS production of the treated groups was determined by comparing to the untreated control.

### Apoptosis analysis

The procedure was performed with Annexin V-FITC Apoptosis Detection Kit (BD Pharmingen, USA) using a modified protocol by Rieger *et al.* [27]. Briefly, upon completion of treatments, cells were harvested and washed with binding buffer. Cells were counted to obtain a final concentration of 1 × 10^6^ cells/ml. Then Annexin V and propidium iodide (PI) were added and incubated in dark for 15 minutes. After washing, cell suspension was fixed with 1% formaldehyde for 10 minutes on ice. Subsequently, washing was performed twice with binding buffer followed by addition of RNase (EMD Biosciences, USA) which then incubated for 15 minutes at 37°C. Finally, samples were washed and ready for analysis with FACSCalibur flow cytometer (BD Biosciences, USA) and the software Cell Quest Pro.

### Mitochondrial membrane potential measurement

Mitochondrial membrane potential (MMP or ΔΨm) is an important indicator of mitochondrial functionality. Apoptosis through mitochondrial-mediated pathway can be assessed by performing MMP assay using MitoScreen kit (BD Pharmingen, USA) according to the protocols provided. Briefly, cells were collected by trypsinization and centrifugation. Washing with PBS was carried out and cells were counted to obtain a final concentration of 1 × 10^6^ cells/ml. Working staining solution was prepared from the JC-1 powdered dye and assay buffer at a ratio of 1:99, which was then added to the cells. Incubation was carried out at 37°C in 5% CO_2_ incubator for 15 minutes. Cells were washed twice in assay buffer before analyzed with flow cytometer. Mitochondrial depolarization is indicated by a decrease in the red JC aggregates/green JC monomer fluorescence intensity ratio.

### Caspase-3 detection

Caspase-3 is a proteolytic enzyme activated during apoptosis. It was detected using FITC active caspase-3 apoptosis kit (BD Pharmingen, USA) according to the protocol provided. Cells were first collected by trypsinization and centrifugation. Then, cells were washed with PBS twice and resuspended in BD Cytofix/ Cytoperm™ fixation and permeabilization solution at 1 × 10^6^ cells/0.5 ml. Subsequently cells were incubated on ice for 20 minutes. Fixation solution was discarded after centrifugation and cells were washed twice with 0.5 ml of BD Perm/Wash™ buffer. A hundred microliters of BD Perm/Wash™ buffer and 20 μl of FITC anti-active caspase-3 antibody were added to each sample and incubated for 30 minutes at room temperature. Then, cells were washed with 1 ml of BD Perm/Wash™ buffer. Finally cells were resuspended in 0.5 ml of the same buffer and transferred to FACS tube for flow cytometry analysis.

### Statistical analysis

Data was collected as triplicate from at least 3 independent experiments. The results were expressed as mean ± standard deviation. Statistical significance was assessed with Student’s t-test. P value <0.05 was considered significant.

## Results & Discussion

### Cytotoxic profile of EBN extracts (S1 and S2)

Toxicity study was first performed with addition of EBN extracts to SH-SY5Y cells to determine the concentration-wide effect as well as MNTDs of the extracts on neuronal culture. MNTD is the maximal dose just below the threshold for cell toxicity that demonstrates no cytotoxic effect. Half of the MNTD value was also determined in order to study the effect of EBN treatment at lower concentration.

**Figure 1:**
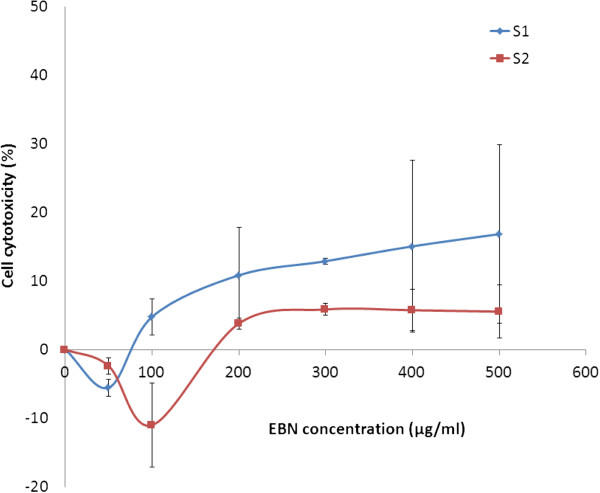
**Cytotoxic effect of EBN extracts on SH-SY5Y cells.** Cytotoxicity percentage of SH-SY5Y cells upon 48 hours treatment with either S1 or S2 was tested across a wide range of concentration from 0 to 500 μg/ml and MTT assay was carried out. Maximum non-toxic dose was then determined from the graph. The data shown are means ± S.D. of three independent experiments performed in triplicates.

In the graph of cytotoxicity percentage in SH-SY5Y cells against EBN extract concentration (Figure 1), it was found that there was an increasing trend of cytotoxicity along with the concentration. However, cell death was not evident at concentration below 100 μg/ml for S1 and 200 μg/ml for S2. MNTDs are the concentrations at which cytotoxicity starts to become evident (where line touches x-axis). As determined from the graph, MNTDs were 76.25 ± 16.52 μg/ml for S1 and 150 ± 36.06 μg/ml for S2. Meanwhile the ½ MNTDs were 38.13 μg/ml and 75 μg/ml for S1 and S2, respectively. Overall the cytotoxicity of S1 was double as much as the cytotoxicity of S2. Such discrepancy could be due to varying methods employed in preparing the two extracts. In fact, compound solubility and stability are major factors that contribute to varied activities in different extracts [28]. S2 was extracted with water thus one might expect the resulting sample to contain only water-soluble substances. Also, high temperature applied during the water extraction process might have affected the potency of proteins within the EBN, possibly through denaturation. Based on the observations, it is likely that the water-soluble substances possess less cytotoxic effect comparing to S1, the crude EBN extract.

### EBN extracts prevent 6-OHDA-induced apoptotic changes in SH-SY5Y cells

**Figure 2:**
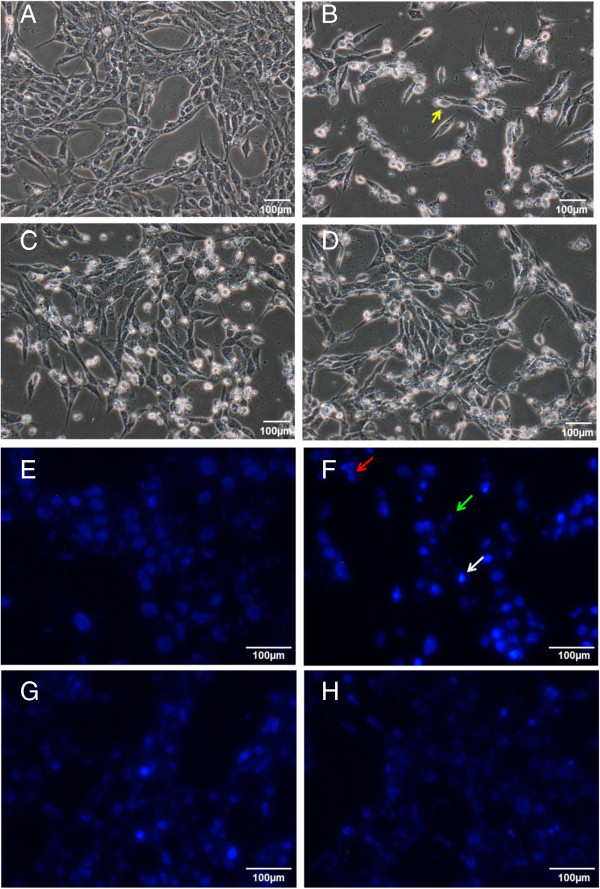
**Effect of EBN extracts on morphological and nuclear changes of 6-OHDA-challenged SH-SY5Y cells.** Microscopic images were taken after 48 hours of treatment. Figures **A-D** are bright field images while Figures **E-H** are fluorescent images taken after Hoechst 33258 staining. Figures **A** and **E**: control group; Figures **B** and **F**: 6-OHDA group; Figures **C** and **G**: S1 MNTD + 6-OHDA-treated group; Figures **D** and **H**: S2 MNTD + 6-OHDA-treated group. Cell shrinkage is indicated by cell losing its distinctive neuronal shape and has becomes smaller in size (yellow arrow in Figure 2B), DNA fragmentation is indicated by cluster of nuclei fragments (red arrow in Figure 2F), shrunken cell is indicated by smaller and distorted nuclei (green arrow in Figure 2F) while nuclear chromatin condensation is indicated by brightly fluorescent nuclei (white arrow in Figure 2F).

A number of cellular morphological changes including cytoplasmic condensation resulting in reduced cell size, plasma membrane undulations or blebbing, condensation of chromatin at nuclear periphery, dilatation of endoplasmic reticulum and formation of apoptotic bodies represent the typical characteristics of apoptosis [29, 30]. In the present study, changes in cellular morphology of SH-SY5Y upon different treatments were assessed by microscopic examination. Untreated SH-SY5Y cells had a distinctive neuronal shape with typical long neurite outgrowth. In addition, cell membrane was intact and there were minimal dead cells (Figure 2A). Nuclear staining with DNA-binding fluorescent dye Hoechst 33258 showed homogenously stained regular rounded nuclei in control cells (Figure 2E). However, when incubated with 100 μM 6-OHDA (an optimum concentration determined from our studies earlier; Data not shown), cell death was made evident by the presence of shiny, floating and round-shaped cells under bright field microscopy (Figure 2B). Shrinking cells which gradually lost their elongated neuronal shape and have shrunken in size were also detected (yellow arrow in Figure 2B). Meanwhile, increased number of bright fluorescent nuclei indicative of chromatin condensation (white arrow in Figure 2F), as well as nuclear fragmentation (red arrow in Figure 2F) were apparent after Hoechst staining. Smaller asymmetrical nuclei were also seen as a result of cell shrinkage (green arrow in Figure 2F). These features altogether suggest that 6-OHDA-induced SH-SY5Y cell death was likely to be mediated through apoptosis. This finding is supported by previous study which concluded that the selective catecholaminergic neurotoxin induces oxidative stress-associated cell death primarily through apoptosis [31].

Nonetheless, pre-treatment with S1 or S2 for 24 hours prior to the addition of 6-OHDA conferred protection to SH-SY5Y cells by reducing cell death in the culture (Figure 2C-D). In addition, the nuclear apoptotic changes induced by 6-OHDA were less noted in the cells after pre-treatment with S1 and S2 (Figure 2G-H), suggesting that EBN may be effective in reversing the cytotoxic effect of 6-OHDA.

### S1 improves cell viability in 6-OHDA-challenged SH-SY5Y cells

**Figure 3:**
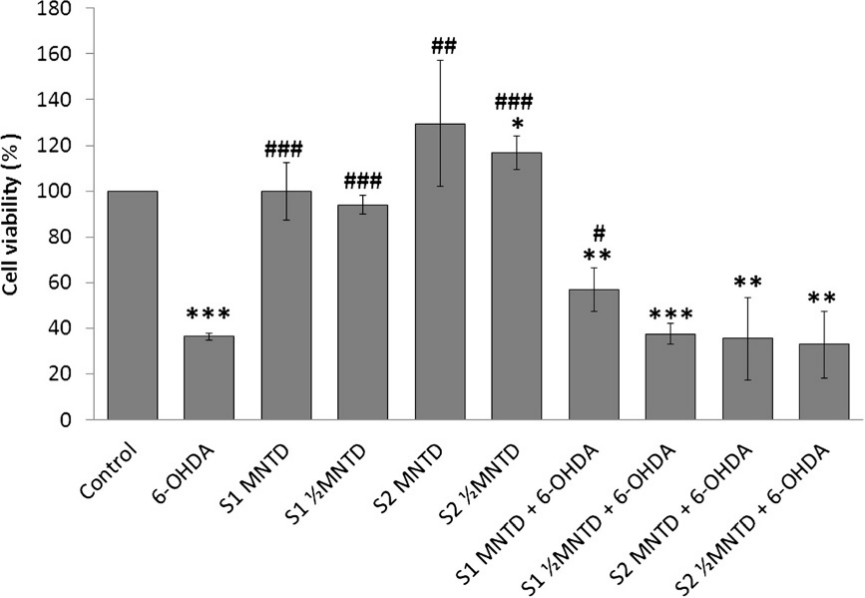
**Effect of EBN extracts on 6-OHDA-challenged SH-SY5Y cell viability.** Cell viability was assessed with MTT assay and data shown are means ± S.D. of three independent experiments performed in triplicates. *P < 0.05; **P < 0.01; ***P < 0.001 versus untreated control cells while ^#^P < 0.05, ^##^P < 0.01; ^###^P < 0.001 versus 6-OHDA treated cells.

To quantify the cell viability from those observed in the morphological study, MTT assay was performed. Upon challenge with 100 μM 6-OHDA for 24 hours, cell viability decreased significantly to about 40% of that of control (Figure 3). Pre-treatment with EBN extracts followed by co-incubation with 6-OHDA generally did not improve cell viability except in the cells treated with S1 at respective MNTD. About 20% increase in the cell viability was observed under that treatment as compared to the 6-OHDA group. This could be due to mitogenic property of S1 that promoted cell growth, as made evident by a study by Zainal Abidin *et al.* which shows that EBN promoted cell division in rabbit corneal keratocytes [32]. Moreover, acid hydrolysates of EBN have been shown to promote proliferation of human colonic adenocarcinoma (Caco-2) cells [33]. The same report also pointed out that sialic acid treatment alone induced significant Caco-2 proliferation. Taken together the finding by Yagi *et al.* which showed that sialic acid was present in EBN, it is suggested that sialic acid could be the bioactive compound that we are interested in [17].

On the contrary, S2 in overall did not prevent cell death, which may be explained by the lower activity associated with the water extract discussed earlier. Meanwhile, EBN treatment alone for 48 hours did not affect cell survival, indicating that EBN treatments at MNTDs were non-cytotoxic to SH-SY5Y cells.

### S2 attenuates ROS build up in 6-OHDA-challenged SH-SY5Y cells

**Figure 4:**
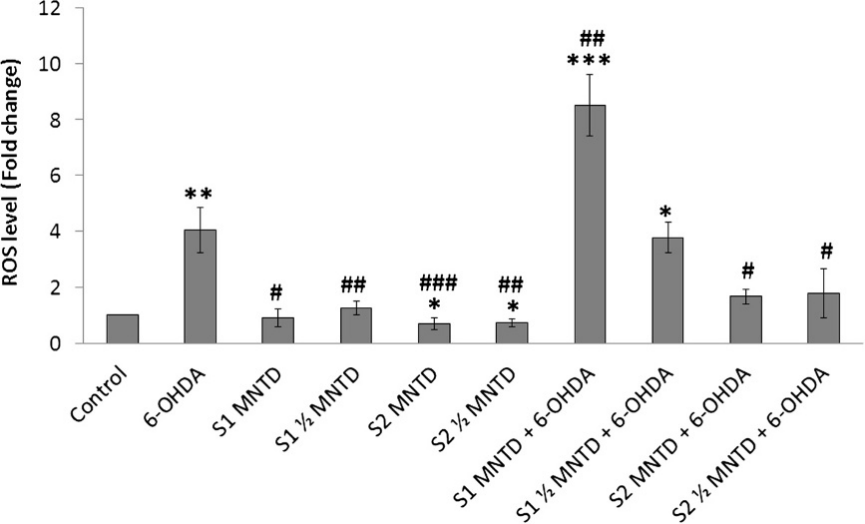
**Effect of EBN extracts on intracellular reactive oxygen species (ROS) production in 6-OHDA-challenged SH-SY5Y cells.** Intracellular ROS levels of treated groups were assessed with DCFH-DA assay and data shown are means ± S.D. of three independent experiments performed in triplicates. *P < 0.05; **P < 0.01; ***P < 0.001 versus untreated control cells while ^#^P < 0.05, ^##^P < 0.01; ^###^P < 0.001 versus 6-OHDA treated cells.

The overall oxidative status in SH-SY5Y cells was assessed with the DCFH-DA assay. Figure 4 showed that ROS was maintained at basal level when SH-SY5Y cells were treated with EBN extracts alone, indicating that EBN alone did not induce oxidative stress within the cell. Furthermore, S2 caused significant drop in ROS production suggesting the protective role of EBN as a free radical species scavenger.

Intracellular ROS production was augmented by 4 fold upon 6-OHDA exposure when compared to control group. Pre-treatment with S1 at high dose (MNTD) did not restore ROS to the basal level, but instead promoted intracellular ROS build up twice as much as the level of that in cell treated with 6-OHDA alone. This could be due to the presence of reactive compounds in S1 which prompted the generation of extra free radical in cells on top of the existing oxidative load, for example a 66 kDa major allergen protein in EBN [34]. As allergenic immune reaction is often linked to increased intracellular ROS production, this putative protein possesses homology to a domain of an ovoinhibitor precursor in chicken could possibly be the contributor of intracellular ROS load in S1-treated SH-SY5Y cells [35, 36].

Meanwhile, ROS production was significantly attenuated to sub-control level by S2 treatment, in which high dose seemed to suppress ROS production better than low dose. These findings suggest that the water-soluble compounds in S2 could be more effective in scavenging intracellular ROS if present in high dose, whereas in crude extract high dose would do the opposite effect. The difference in activities of S1 and S2 can be inferred from the different methods used in extract preparation. Although both extracts derived from the same raw material, solubility and solvent accessibility of bioactive compounds in crude and water extracts may affect their chemical properties and hence, the bioactivities. In particular, water extraction method involves heat treatment at 100°C and therefore may modify tertiary conformational structure of the native EBN protein. This manipulation could possibly uncover the nucleophilic amino acid residues that present in the protein core, such as cysteine’s sulfhydryl groups [37]. These amino acid residues are free radical scavenger because they are oxidized preferentially to the membrane phospholipids. S2 may therefore possess higher antioxidant potential than S1 because of greater solvent exposure of free radical scavenging amino acid residues as a result of heat-assisted protein denaturation.

Generally, reduction of ROS level would ameliorate oxidative stress-related cellular damage and cell death, but this was not observed in the present study. Results from ROS measurement when put together with MTT results raised an intriguing question because S1 had successfully improved cell viability although it triggered ROS generation. One explanation for this is that S1 could have initiated cytoprotective mechanism other than direct ROS-scavenging, such as the promotion of antioxidant defense system through nuclear erythroid 2-related factor 2 - antioxidant responsive element (Nrf2-ARE) signaling [38]. It has been suggested that exogenous protein can act through Nrf2-ARE signaling pathway to induce expression of endogenous antioxidant enzymes [39]. In fact, ARE activation have been demonstrated to be protective against *in vitro* cell death induced by dopamine and 6-OHDA, likely due to enhanced expression of the antioxidant proteins such as glutathione S-transferase A2, heme oxygenase-1 and NAD(P)H quinoneoxidoreductase 1 [40–42]. Interestingly, Nrf2-ARE activation is a redox-sensitive process thus this process can be triggered in response to intracellular oxidative changes, as seen in the action of apomorphine whereby ROS produced by the drug itself acts as Nrf2-ARE pathway activator [43, 44]. Therefore, it is possible that S1, in the presence of ROS, generates signals that initialize molecular changes to result in cytoprotection and hence improved cell viability. However, further works should be done to confirm the role of these members of the intracellular antioxidant response system in the neuroprotective mechanism of S1. Investigations on expression of the antioxidant genes or proteins, and nuclear translocation of Nrf2 protein could be performed in the future.

### EBN extracts reduce early apoptotic event in 6-OHDA-challenged SH-SY5Y cells

**Figure 5:**
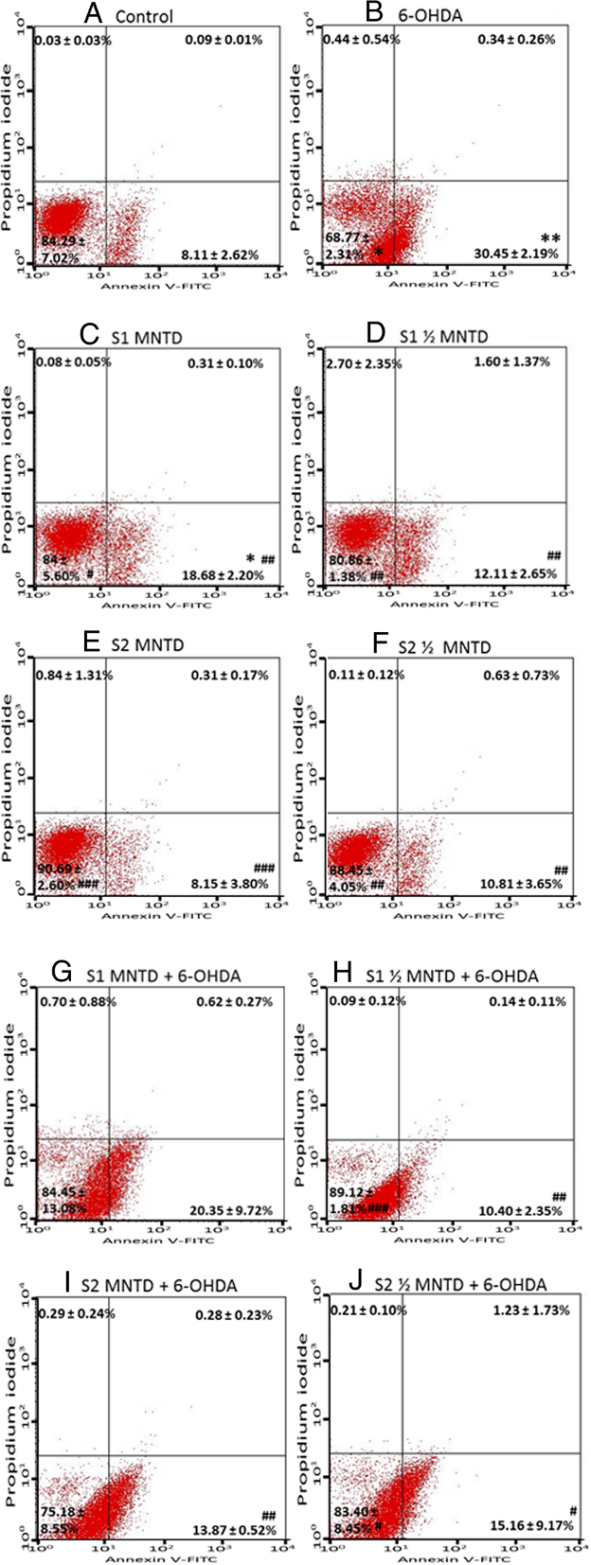
**Effect of EBN extracts on 6-OHDA-induced apoptosis in SH-SY5Y cells.** Cells treated with EBN extracts for 48 hours were analyzed by Annexin V-propidium iodide double staining. Representative plots of propidium iodide versus Annexin V-FITC fluorescence signals are shown **(A-J)**. Results shown are the mean ± S.D. of three independent experiments. *P < 0.05; **P < 0.01 versus untreated control cells while ^#^P < 0.05, ^##^P < 0.01; ^###^P < 0.001 versus 6-OHDA treated cells.

Apoptotic event was investigated by Annexin V-PI double staining method to identify the mode of cell death. Several previous reports showed that apoptosis of dopaminergic neurons was found to increase with 6-OHDA-induced oxidative stress [45–48]. In this assay, cells residing at different stages of apoptosis upon exposure to 6-OHDA were identified by differential staining of membrane phosphatidylserine and DNA, whereby cells are grouped and represented in dot plot as healthy (lower left quadrant), early apoptotic (lower right quadrant), late apoptotic (upper right quadrant) and necrotic (upper left quadrant) ones. Early apoptosis, represented by cell stained positively with Annexin V due to phosphatidylserine translocation towards outer membrane surface followed by loss of membrane integrity, was found to be the major cell death mechanism in cell treated with 6-OHDA for 24 hours. It accounted for about 30% of the cell population (Figure 5B, lower right quadrant), which is in concordance with other reports [49, 50].

Generally EBN treatment alone did not stimulate apoptotic event (Figure 5D-F), but it effectively reduced early apoptotic injury in cells challenged with 6-OHDA (Figure 5H-J). The results suggest that EBN is a potential neuroprotective agent which acts by inhibiting apoptosis. However, S1 treatment increased the percentage of apoptotic population in normal culture (Figure 5C) and did not reduced the early apoptosis induced by 6-OHDA (Figure 5G) when given at MNTD. Such findings imply that high dose of crude extract did not improve apoptosis and could have itself contributed to apoptosis in SH-SY5Y cells, despite the fact that MNTD used for S1 treatment had been pre-determined as non-toxic. The apoptosis-inducing nature of S1 at MNTD may be related to the elevated intracellular ROS produced as determined from the DCFH-DA assay previously.

### EBN extracts did not improve mitochondrial dysfunction in 6-OHDA-challenged SH-SY5Y cells

**Figure 6:**
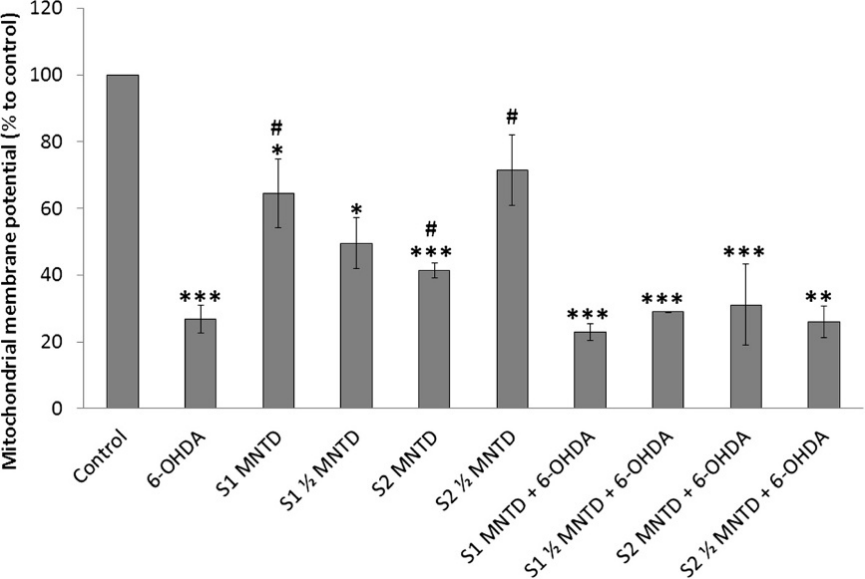
**Effect of EBN extracts on mitochondrial membrane potential (MMP) in 6-OHDA-challenged SH-SY5Y cells.** MMP was assessed with mitochondria-selective JC-1 dye and results shown are the mean ± S.D. for three independent experiments. *P < 0.05; **P < 0.01 versus untreated control cells while ^#^P < 0.05 versus 6-OHDA treated cells.

Mitochondrial dysfunction as defined by collapse in MMP is an early and critical event in cellular apoptosis. In fact, the opening of mitochondrial transition pore upon different stimuli precedes the depolarization of mitochondrial potential, subsequently leading to increased mitochondrial permeability and thus the efflux of pro-apoptotic factors such as cytochrome c and pro-caspases [51]. The MMP in SH-SY5Y cells was measured in order to study the alteration in mitochondrial activity during apoptosis. In the present study, MMP of SH-SY5Y cells dropped drastically to 27% of that in control when the cell was exposed to 100 μM 6-OHDA for 24 hours (Figure 6). Although the action through which 6-OHDA induces cytotoxicity in neuronal cell lines such as SH-SY5Y and PC12 has been linked to ROS outburst, literature is available to show that failure of cellular respiratory complex may be the direct cause of apoptosis. Gomez- Lazaro *et al.* found that mitochondrial fragmentation constitutes early event in mitochondrial dysfunction and eventually cell death of SH-SY5Y. The author further revealed that 6-OHDA-induced SH-SY5Y cell death was reversible by blockage of the mitochondrial fission activity [51]. Taken together, the findings support the indispensable role of mitochondrial integrity in SH-SY5Y cells’ survival. In fact, studies have successfully demonstrated neuroprotection against apoptosis via restoration of mitochondrial functionality by natural products, such as the herbal medicine Chunghyuldan, which ameliorated PD-like behavioral symptoms by preserving dopaminergic neurons in the nigrostriatal region of PD mice model [52].

Yet, mitochondrial functionality in the intoxicated cell was not improved with EBN treatment in the present study. There was no significant difference in MMPs between the EBN-co-treated and 6-OHDA groups. Therefore, the cell-promoting effect of S1 seen in MTT assay may be not related to resuscitation of MMP in 6-OHDA-challenged SH-SY5Y cells. Although 6-OHDA-induced cytotoxicity is mainly associated with mitochondrial respiratory dysfunction, an opposite mitochondrial-independent pathway is as well indicated in a number of studies [53, 54]. As such, involvement of non-mitochondrial mechanism may be implicated in the neuroprotection conferred by the EBN crude extract.

Notably, EBN treatment alone was shown to bring down the level of MMP significantly, indicating that the addition of foreign compound to the SH-SY5Y culture could affect the mitochondrial status. Still, treatment with EBN extracts alone for 48 hours did not exhibit any detrimental effect on the SH-SY5Y’s cell viability and oxidative status.

### S2 inhibits cleavage of caspase-3 in 6-OHDA-challenged SH-SY5Y cells

Caspase-3 is the executioner protein of the apoptotic process and remains as inactive procaspase until it is being cleaved by activated initiator caspases such as caspase-8 or caspase-9. Its activation leads to downstream mechanisms which involve poly(ADP-ribose) polymerase-mediated DNA cleavage and breakdown of proteins essential for maintenance of cytoskeletal structure. Eventually, activation of caspase-3 results in DNA fragmentation and apoptosis [55].

**Figure 7:**
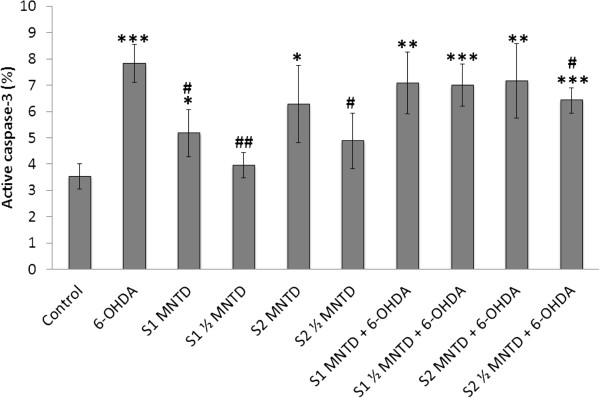
**Effect of EBN extracts on cleavage of caspase-3 in SH-SY5Y cells challenged with 6-OHDA.** The release of active caspase-3 into cytosol was assessed by immunostaining using FITC-conjugated antibody and results were generated from flow cytometry. Results shown are the mean ± S.D. of three independent experiments. **P < 0.05; **P < 0.01 versus untreated control cells while ^#^P < 0.05, ^##^P < 0.01versus 6-OHDA treated cells.

Elevated active caspase-3 level was detected in SH-SY5Y cells treated with 6-OHDA, which was 7.8% as compared to 3.5% in control (Figure 7). However, S2 at ½ MNTD managed to attenuate caspase-3 activation in the cell. This explains the parallel reduction in early apoptotic population by S2 at ½ MNTD as seen from Annexin V-PI analysis, thus consolidates the role of caspase-3 activation in the process of 6-OHDA-induced SH-SY5Y apoptosis [56].

Caspase-3 can be activated through two machineries, namely the mitochondria-related apoptosis protease-activating factor-1/caspase-9/caspase-3 cascade, and Fas-associated adapter protein/caspase-8/caspase-3 cascade which is the extrinsic apoptotic pathway [57]. Based on the observation from MMP assay in which S2 failed to resuscitate mitochondrial depolarization in SH-SY5Y cells challenged with 6-OHDA, it is likely that S2 inhibited caspase-3 through modulation of the extrinsic pathway which has little relation to the mitochondrial activity. However, future study is warranted to investigate the involvement of extrinsic pathway in S2-mediated neuroprotection. For EBN treatment only, both S1 and S2 when given at MNTD induced release of active caspase-3, signifying that high dose of the extracts may trigger early apoptosis event and hence lower concentrations should be used for the treatment.

### Remarks on discordance between cytotoxicity and apoptotic parameters

Current finding showed that S1 treatment at MNTD improved SH-SY5Y cell viability despite being unable to prevent apoptosis and restore ROS and caspase-3 to basal level. On the contrary, S2 treatment has successfully attenuated ROS development and reduced apoptosis and caspase-3 release, yet it failed to reverse cytotoxic effect of 6-OHDA. This is intriguing as cytotoxicity is positively associated with apoptotic event, oxidative stress and caspase-3 activation. However, it is worth mentioning that cytotoxicity can be mediated by several different cell death mechanisms. Other than apoptosis, cell death can also be orchestrated by autophagy and necrosis [58]. Under such circumstances, cells are likely to either slowly sequestrated and then degraded by autolysomes through autophagy, or undergo necrosis upon activation of death domain receptors on cell surface. Cytotoxicity is thus attributed, but not exclusively, to apoptosis parameters such as externalization of phosphatidylserine on plasma membrane and activation of caspase signaling cascade. Meanwhile caspase-dependent cell death mechanism has often been described as cardinal to 6-OHDA-induced apoptosis in SH-SY5Y cells, it is proposed that another caspase-independent mechanism may also exist in a cell death scenario [59]. Such claim is supported by literatures, stating that in dying cells, caspase inhibition alone does not necessary grant full resuscitation from apoptosis but may switch cell death to alternative autophagic or necrotic modes instead [60, 61]. In fact, dysregulation of autophagic response in the neurons has been linked to PD incidence [62] and autophagic changes in SH-SY5Y cells are inducible with 6-OHDA treatment [63]. On the other hand, oxidative burst from 6-OHDA could contribute to membrane rupture and therefore necrosis in neurons too [64]. Considering that autophagy or other cell death mechanisms could be activated and participated in regulating cell death in our PD cell model, hence it is indefinite that inhibition of apoptosis or caspase activation by S2 treatment would see corresponding decrease in cell death or cytotoxicity. This may partly explain the failure of S2 extract to counteract cytotoxicity due to 6-OHDA exposure regardless of its ability to attenuate apoptosis and caspase-3 cleavage.

## Conclusions

It has been successfully demonstrated that EBN extracts confer neuroprotection in 6-OHDA-challenged SH-SY5Y cell model. Particularly, S1 demonstrated neuroprotective potential by improving cell viability while S2 inhibited oxidative stress and caspase-3 activation. Both the EBN extracts shown to inhibit apoptosis. Further investigation is needed to identify and elaborate the properties of the bioactive compounds in EBN that are responsible for the neuroprotective benefits. In summary, the present study suggests that EBN may be effective in the treatment of neurodegenerative diseases where oxidative stress plays a causal role.

## Abbreviations

6-OHDA: 6-hydroxydopamine
ARE: Antioxidant responsive element
Caco-2: Human colonic adenocarcinoma
DCF-DA: 2′ 7′-dichlorofluorescein diacetate
DMSO: Dimethyl sulphoxide
EBN: Edible bird’s nest
EGF: Epidermal growth factor
MMP: Mitochondrial membrane potential
MNTD: Maximum non-toxic dose
MTT: 3-(4 5-dimethylthiazol-2-yl)-2, 5-diphenyltetrazolium bromide
Nrf2: nuclear erythroid 2-related factor 2
PBS: Phosphate-buffered saline
PD: Parkinson’s disease
PI: Propidium iodide
ROS: Reactive oxygen species
SH-SY5Y: Human neuroblastoma cell.
